# Delayed Emergence Due to Severe Respiratory Acidosis Following Prolonged Spine Surgery: A Case Report

**DOI:** 10.7759/cureus.109051

**Published:** 2026-05-17

**Authors:** Imran Ahmed Khan, Jai Prakash Tiwari

**Affiliations:** 1 Anesthesiology and Public Health, Keshav Memorial Charity (KMC) Medical College and Hospital, Maharajganj, IND; 2 Anaesthesia and Critical Care, Rajarshi Dashrath Autonomous State Medical College, Ayodhya, IND

**Keywords:** delayed emergence, general anesthesia, hypercapnia, postoperative care, respiratory acidosis, spine surgery

## Abstract

Delayed emergence from anesthesia is a frequent challenge in surgical settings. Common etiologies include residual drug effects, metabolic derangements, or neurological insults. We report a case of a 55-year-old woman who underwent an elective six-hour lumbar laminectomy and cervical implantation surgery. Despite an uneventful intraoperative course and routine neuromuscular blockade reversal, the patient exhibited somnolence and ventilatory failure after reversal and extubation. Arterial blood gas (ABG) analysis revealed severe respiratory acidosis. The patient required re-intubation and controlled mechanical ventilation to facilitate carbon dioxide (CO₂) washout. Following normalization of arterial partial pressure of carbon dioxide (PaCO₂), the patient regained consciousness and was successfully extubated the following morning. This case highlights the importance of monitoring ventilation adequacy in the transition from controlled to spontaneous respiration following long-duration surgeries. Importantly, normal intraoperative end-tidal CO₂ values should not be considered reassuring in high-risk patients, as significant arterial hypercapnia may remain undetected.

## Introduction

Emergence from general anesthesia (GA) is often expected to be a predictable process. When a patient fails to regain consciousness or maintain adequate ventilation within 30-60 minutes of discontinuing anesthetic agents, it is classified as delayed emergence [1]. It may occur due to pharmacological causes (residual anesthetics, opioids, or neuromuscular blockade), metabolic disturbances (hypoglycemia, hypercapnia, or electrolyte imbalance), or neurological events (stroke, seizure, or hypoxic injury) [2]. In prolonged spine surgeries, factors such as prone positioning and significant opioid titration can complicate the respiratory drive [3]. Postoperative hypercapnia and carbon dioxide (CO₂) narcosis represent important, potentially reversible metabolic causes of delayed emergence, particularly in high-risk patients. In such patients, normal intraoperative end-tidal CO₂ (EtCO₂) values may not reliably reflect arterial partial pressure of carbon dioxide (PaCO₂) due to ventilation-perfusion mismatch or increased physiologic dead space, potentially masking significant arterial hypercapnia until extubation. Severe hypercapnia following apparently adequate intraoperative ventilation can be managed effectively if the primary cause is diagnosed early [4]. This report discusses a case of severe respiratory acidosis resulting from CO₂ narcosis, mimicking a delayed emergence from anesthesia.

## Case presentation

A 55-year-old female patient with an American Society of Anesthesiologists Physical Status (ASA-PS) II [5] and a body mass index of 34 kg/m² was scheduled for a combined lumbar laminectomy (L3/4 and L4/5) and cervical implant (C4/5 and C5/6). Her preoperative evaluation was unremarkable, except for a history of chronic obstructive pulmonary disease (COPD). The patient had no history of obstructive sleep apnea. Her preoperative laboratory investigations, including hematology and biochemistry, were within normal limits, and electrocardiography (ECG) was normal. Written informed consent was obtained from the patient for the publication of this case report with due precaution to keep anonymity. The manuscript adheres to the standard case reporting (CARE) guidelines.

Intraoperative management

**Figure 1:**
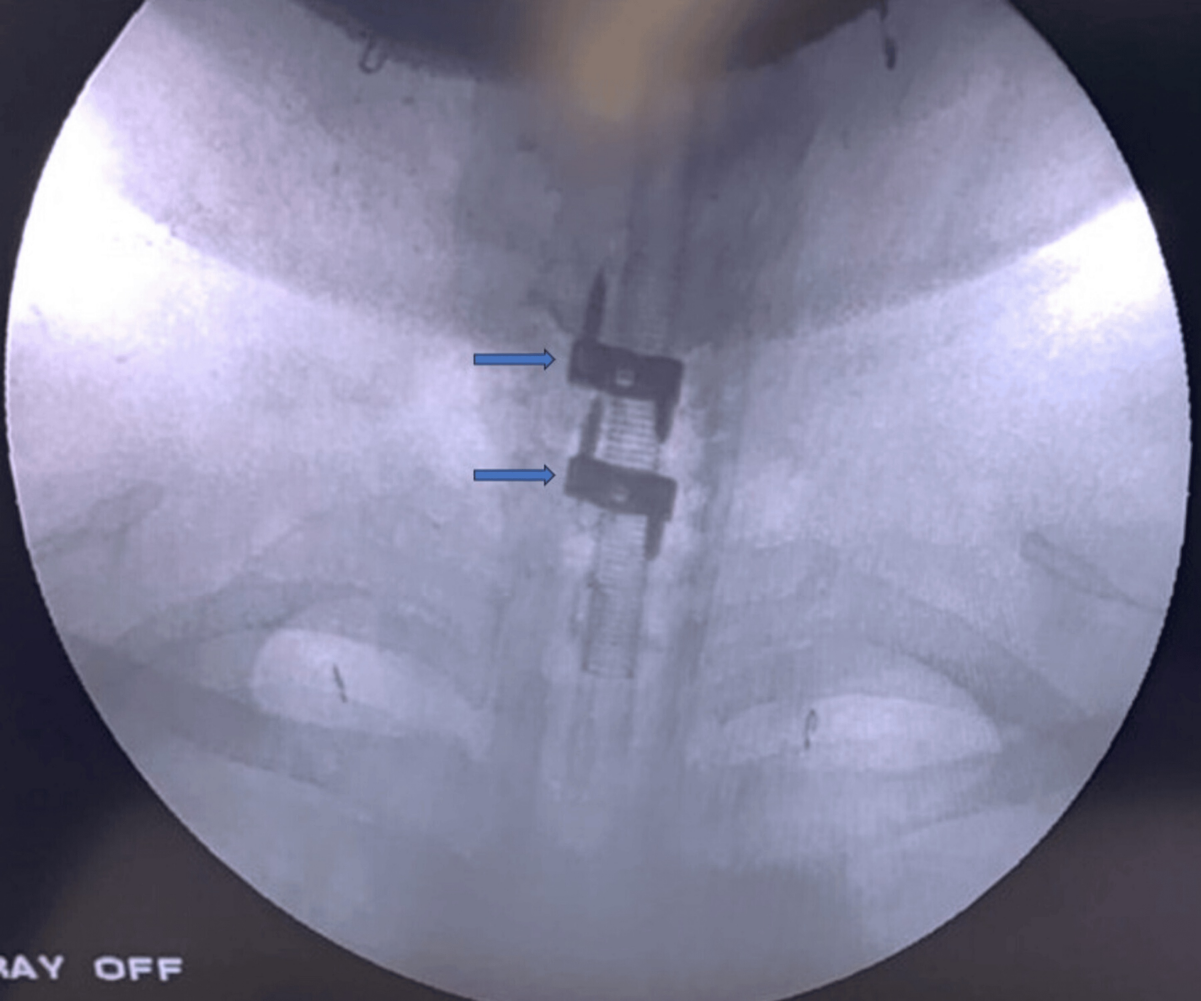
Postoperative X-ray of the cervical spine showing implants at C4/5 and C5/6 (blue arrows)

The patient was fasted overnight and shifted at 9.00 a.m. to the operating theater. Patient identity was verified, and routine monitors comprising a pulse oximeter, noninvasive blood pressure, a five-lead ECG, EtCO₂, and a temperature probe were applied, and their function verified. Two 18-gauge cannulas were inserted on the dorsum of the hand, one on each side. A preemptive analgesia was provided intravenously over 20 minutes using a multimodal analgesic protocol, including paracetamol (1 g), tramadol (25 mg), magnesium sulfate (1 g), and 80 mg of preservative-free lidocaine [6]. A 25 mg bolus of ketamine with 0.2 mg glycopyrrolate and 1 mg midazolam was also given intravenously. Anesthesia was induced with propofol (90 mg) and vecuronium (5 mg); the airway was secured with a 7.0 internal diameter flexometallic endotracheal tube and secured with an adhesive tape. Anesthesia was maintained with isoflurane in an oxygen-nitrous mixture and vecuronium infusion. The patient was put on mechanical ventilation with a 500 ml tidal volume and a rate of 14 per minute. Injection fentanyl 50 µg, dexamethasone 8 mg, and injection diclofenac potassium 75 mg were then injected as part of multimodal analgesia. The patient was placed in the prone position, and the lumbar procedure was completed. Afterward, the patient was made supine to proceed with the cervical procedure through the anterior approach as per the plan (Figure 1). The image confirms the surgical procedure performed via an anterior approach but is not central to the pathophysiology of delayed emergence. The total operative time was about six hours. The patient was hemodynamically stable with temperature and EtCO₂ in the normal range (mean EtCO₂ 32-38 mmHg). Blood loss was minimal, and fluid resuscitation was appropriate. Vecuronium infusion was turned off about 30 minutes before the expected completion of the procedure. Isoflurane was tapered and then turned off during the start of the closure. Injection of paracetamol (1 g) was repeated before reversal. Injection ondansetron (4 mg) was given intravenously.

Emergence and crisis

Upon completion of the surgery, inhalational anesthetics were tapered and closed, and the patient was ventilated with 100% oxygen. The residual neuromuscular blockade was reversed with standard doses of neostigmine 2.5 mg and glycopyrrolate 0.5 mg upon verifying pharyngeal reflexes and limb movements on command. Oropharyngeal suction was done, and the patient was extubated at about 3.00 p.m. after meeting standard extubation criteria and put on oxygen through Hudson’s mask at four liters per minute. Although the patient showed some initial effort, she remained sedated, exhibited inadequate tidal volumes, and was unable to maintain peripheral oxygen saturation (SpO₂) of 90% without positive pressure support via the breathing circuit. Subsequently, the patient developed increased blood pressure (160/100 mmHg) and heart rate (130/minute) with bilateral sluggishly reacting pupils. An injection of furosemide 20 mg was given. Despite 60 minutes of supportive care and observation in the operating room, the patient’s mental status and respiratory effort did not improve. She remained bradypneic and obtunded. The operating neurosurgeon, anesthesiologist, and physician were continuously involved in the management process. Her blood sugar was 325 mg/dL. An urgent arterial blood gas (ABG) was performed, revealing the following: pH, 6.8; PaCO₂, 162 mmHg; partial pressure of oxygen (PaO₂), 101.6 mmHg (on supplemental O₂); and bicarbonate, 24.9 mmol/L (Table 1).

**Table 1:** ABG 60 minutes after completion of surgery and extubation showing severe acute respiratory acidosis (pH 6.793, PaCO₂ 162.2 mmHg) ABG, arterial blood gas; pH, logarithmic hydrogen ion concentration; PaCO₂, partial pressure of carbon dioxide; PaO₂, partial pressure of oxygen; HCO₃⁻, bicarbonate; BE, base excess; Na⁺, sodium; K⁺, potassium; Ca⁺⁺, calcium; Cl⁻, chloride; AGap, anion gap; Hct, hematocrit; Glu, glucose; Lac, lactate; BUN, blood urea nitrogen; Creat, creatinine

Parameter	Result	Reference Range	Unit
pH	6.793	7.350-7.450	
PaCO₂	162.2	35.0-48.0	mmHg
PaO₂	101.6	83.0-108.0	mmHg
HCO₃⁻	24.9	21.0-28.0	mmol/L
BE	-9.8	2.0-3.0	mmol/L
Na+	139	138-146	mmol/L
K+	4.3	3.5-4.5	mmol/L
Ca++	1.08	1.15-1.33	mmol/L
Cl-	106	98-107	mmol/L
A Gap	7	7-16	mmol/L
Hct	45	38-51	%
Glu	373	74-100	mg/dL
Lac	2.99	0.56-1.39	mmol/L
BUN	12	8-26	mg/dL
Urea	4.3	2.9-9.3	mmol/L
Creat	1.03	0.51-1.19	mg/dL

The diagnosis of severe respiratory acidosis and CO₂ narcosis was made. The patient was re-intubated to secure the airway and placed on mechanical ventilation (volume control mode) to facilitate controlled CO₂ elimination. An injection of mannitol 100 ml and an injection of methylprednisolone 125 mg were infused empirically while ruling out intracranial pathology. Ten units of subcutaneous regular insulin were injected.

A repeat ABG after four hours of mechanical ventilation showed a pH of 7.36 and a PaCO₂ of 36.9 mmHg (Table 2). Concurrent with the normalization of pH, the patient became conscious, opening her eyes and following simple commands. Hemodynamics became stable. To ensure safety and prevent secondary hypercapnia or exhaustion, the patient was kept sedated on assisted ventilation overnight. At 7 a.m. the following morning, sedation was discontinued. After meeting all weaning criteria, she was successfully extubated at 10 a.m. without further complications. The patient was discharged in a stable condition on the sixth postoperative day.

**Table 2:** ABG showing normalization of pH and PaCO₂ after 4 hours of mechanical ventilation ABG, arterial blood gas; pH, logarithmic hydrogen ion concentration; PaCO₂, partial pressure of carbon dioxide; PaO₂, partial pressure of oxygen; HCO₃⁻, bicarbonate; BE, base excess; Na⁺, sodium; K⁺, potassium; Ca⁺⁺, calcium; Cl⁻, chloride; AGap, anion gap; Hct, hematocrit; Glu, glucose; Lac, lactate; BUN, blood urea nitrogen; Creat, creatinine

Parameter	Result	Reference Range	Unit
pH	7.357	7.35-7.45	
PaCO₂	36.9	35.0-48.0	mmHg
PaO₂	125.4	83.0-108.0	mmHg
HCO₃⁻	20.7	21.0-28.0	mmol/L
BE	-4.8	-5	mmol/L
Na+	136	138-146	mmol/L
K+	4.3	3.5-4.5	mmol/L
Ca++	0.92	1.15-1.33	mmol/L
Cl-	107	98-107	mmol/L
A Gap	10	7-16	mmol/L
Hct	33	38-51	%
Glu	241	74-100	mg/dL
Lac	2.23	0.56-1.39	mmol/L
BUN	14	8-26	mg/dL
Urea	5	2.9-9.3	mmol/L
Creat	1.09	0.51-1.19	mg/dL

## Discussion

This case illustrates the classic presentation of CO₂ narcosis in the postoperative period. While routine reversal was administered, it appears the patient’s minute ventilation was insufficient after reversal to clear the accumulated CO₂ from poor respiratory effort. The markedly elevated PaCO₂ (162 mmHg) with severe acidemia (pH 6.8) observed in this case can be explained by profound alveolar hypoventilation in the setting of multiple predisposing factors, including COPD, obesity, prolonged surgery, and prone positioning. PaCO₂ decreased dramatically from 162.2 to 36.9 mmHg within four hours of controlled ventilation, correlating with rapid neurological recovery. The resulting hypercapnia likely caused central nervous system depression, explaining the patient’s delayed emergence and hypoventilation. High arterial carbon dioxide perioperatively, although uncommon, is well documented in the literature [7,8]. It remains unclear whether significant hypercapnia developed intraoperatively despite apparently acceptable EtCO₂ (32-38 mmHg) or accumulated rapidly after extubation due to inadequate spontaneous ventilation. The absence of intraoperative ABG sampling precludes definitive timing.

Rising PaCO₂ with acidosis depresses the central nervous system, causing fluctuating mental status or unresponsiveness despite anesthetic discontinuation [9]. Carbon dioxide acts as a potent central nervous system depressant at high partial pressures. Typically, PaCO₂ levels above 80 mmHg can induce a "narcosis" effect, where the high levels of CO₂ actually depress the respiratory center rather than stimulating it, creating a vicious cycle of hypoventilation and worsening acidosis [10]. In one case, PaCO₂ rose from 65 to 105 mmHg with worsening mental status until ventilatory support corrected the acidosis and consciousness returned [2].

The prone position can lead to increased intra-abdominal pressure, which elevates the diaphragm and reduces lung compliance. In patients with a high BMI and pre-existing COPD, this reduces alveolar ventilation [11]. We hypothesize that the high PaCO₂ was a result of an undetected EtCO₂-PaCO₂ gradient, potentially exacerbated by the use of nitrous oxide, which can limit the fraction of inspired oxygen (FiO₂) and mask hypoventilation until the point of extubation. Quantitative neuromuscular monitoring (Train-of-Four) was unavailable at our facility, necessitating reliance on clinical recovery markers, which proved insufficient in this complex scenario.

Inhalational agents used during long-duration spine surgery can blunt the hypercapnic ventilatory response, even if the patient appears awake initially. In the transition from the ventilator to the circuit during emergence, if the patient’s spontaneous tidal volumes are low, they may primarily ventilate anatomical dead space, leading to rapid CO₂ accumulation [12]. The patient was not diabetic, but point-of-care testing revealed a blood glucose level of 325 mg/dL. Severe acidosis triggers a massive catecholamine release, which causes hyperglycemia [13]. Injection of furosemide 20 mg was given in view of low urine output.

In this case, we initially considered any cerebrovascular event or residual neuromuscular block. However, phrenic nerve involvement must also be considered a primary differential, particularly following an anterior cervical approach. Traction or compression of the phrenic nerve (C3-C5) can lead to diaphragmatic paralysis, contributing to the severe respiratory acidosis observed [14]. While the patient’s eventual recovery suggests a metabolic etiology (CO₂ narcosis), the potential for transient nerve paresis to initiate the cycle of hypoventilation cannot be ignored. The initial management focused on ruling out residual sedative effects and providing ventilatory support, with the ABG ultimately identifying the underlying metabolic crisis and shifting the management from waiting to active intervention. The rapid normalization of PaCO₂ following controlled ventilation strongly supports the accuracy of the initial ABG and confirms hypercapnic respiratory failure as the primary etiology. The absence of intraoperative ABG monitoring limits the precise identification of the timing of hypercapnia onset.

Although clinical signs (limb movement, pharyngeal reflexes, and sustained head lift) suggested adequate reversal, quantitative train-of-four monitoring was unavailable. Residual neuromuscular blockade from vecuronium infusion, therefore, cannot be entirely excluded and may have contributed to initial hypoventilation. Noninvasive ventilation options such as high-flow nasal cannula (HFNC) or non-invasive ventilation (NIV) were not utilized in this case; their role in preventing re-intubation in similar high-risk COPD patients merits consideration in future practice.

## Conclusions

Clinicians should maintain a high index of suspicion for respiratory acidosis in any patient with delayed emergence following long-duration surgery, regardless of the perceived stability of the intraoperative course, especially those with obesity, COPD, or prone positioning. Early ABG analysis should be considered. Controlled mechanical ventilation to wash out CO₂ is the definitive treatment, and clinicians should not hesitate to re-intubate to prevent further cardiac or neurological compromise. Multidisciplinary coordination should be practiced to obtain good patients.
